# Bioactivity of PEEK GRF30 and Ti6Al4V SLM in Simulated Body Fluid and Hank’s Balanced Salt Solution

**DOI:** 10.3390/ma14082059

**Published:** 2021-04-19

**Authors:** Piotr Prochor, Żaneta Anna Mierzejewska

**Affiliations:** Institute of Biomedical Engineering, Faculty of Mechanical Engineering, Bialystok University of Technology, 15-351 Białystok, Poland; p.prochor@pb.edu.pl

**Keywords:** biomaterials, hydroxyapatite, SBF, HBSS

## Abstract

In recent years, scientists have defined two main paths for orthopedic implant fabrication: searching for new materials with properties closest to natural bone in order to reduce the stress-shielding effect or creating individually adapted geometry of the implant with the use and Rapid Prototyping methods. Therefore, materials such as PEEK GRF30 and Ti6Al4V selective laser melting (SLM) are of interest. They are defined as materials suitable for implants, however, the knowledge of their bioactivity, a feature which is one of the most desirable properties of biomaterials, is still insufficient. Using Simulated Body Fluid and Hank’s Balanced Salt Solution, the bioactivity of PEEK GRF30 and Ti6Al4V SLM was assessed, as well as commercial Ti6Al4V as a reference material. Ten cylindrical samples of each material were prepared and immersed in solutions per period from 2 to 28 days at 37 °C. Optical analysis of the changes on the examined surfaces suggested that right after 2-day crystals with different morphologies were formed on each material. Further analysis of the chemical composition of the altered surfaces confirmed the formation of a calcium phosphate layer on them, however, the Ca/P ratio was slightly different from 1.67. On the basis of the obtained results, it can be concluded that both PEEK GRF30 and Ti6Al4V SLM are characterized by appropriate—comparable to Ti6Al4V—bioactivity.

## 1. Introduction

Osteoinduction is a process which consists of a sequence of reactions leading to the differentiation of stem cells and stimulates them to transform them into osteoblasts [1]. As a result, it leads to the reconstruction of damaged bone tissue. The osteoinductive property of biomaterial result from its bioactivity, related with the ability to build up (in contact with the blood plasma) the hydroxyapatite layer on the surface of the biomaterial. The hydroxyapatite layer is a natural scaffold for cells, stimulating them to differentiate and multiply [2,3,4,5].

Hydroxyapatite [Ca_10_(OH)_2_(PO_4_)_6_)] is calcium orthophosphate—a salt of a tribasic acid orthophosphoric—H_3_PO_4_ with a Ca/P molar ratio equal to 1.667 containing hydroxide groups (OH^−^). Rough hydroxyapatite surface in the tissue environment and at the physiological pH is the most stable form of calcium phosphate, that is why it also creates a surface with the best biocompatible and bioinductive properties [5,6].

In order to recreate the tissue environment in laboratory conditions, simulation fluids such as HBSS (Hank’s Balanced Salt Solution) and SBF (Simulated Body Fluid) are used for this purpose. Both of them contain inorganic components of blood plasma, rich in chloride ions, which in the tissue environment are mainly responsible for the corrosive processes of metallic biomaterials. The chemical composition of these solutions was selected so that the concentration of ions was comparable to the concentration of biological fluids, and the pH value was as close as possible to the pH of human blood [2,4].

Although the mechanical properties of biomedical materials are already widely known, biological properties of some of them are still not clearly defined. As an example, Ti6Al4V titanium alloy may be specified—its properties have been tested in both laboratory and clinical conditions. Despite the content of aluminum and vanadium, it has been used for decades in the production of implants supplementing bone defects [7].

Traditional technologies of producing titanium implants are time-consuming and expensive. Therefore, rapid prototyping (RP) methods are becoming more and more popular—as a result of their unquestionable advantages and possibilities [8,9,10]. These methods rely on additive manufacturing (AM) of nearly ready to use implants, by creating them layer by layer. Among RP methods selective laser melting (SLM) can be distinguished. It allows manufacturing implants with complex geometry by selectively consolidating successive layers of powdered material with the use of thermal energy provided by a focused and computer-controlled laser beam. Powdered Ti6Al4V, after melting, is characterized by significant porosity and different mechanical and biological properties in relation to the material cast or shaped by forging [11,12,13,14,15]. And although the mechanical properties of Ti6Al4V SLM are often reported in the literature, its biological properties, including bioactivity, are not clearly defined. This makes it necessary to study the still undefined properties of Ti6Al4V SLM and to compare this material to its conventional counterpart in terms of the ability to form a hydroxyapatite layer on the surface of the implant.

Although PEEK has been explored for a number of medical applications, especially for clinical dentistry, it has very limited osteoconductive properties. Current research indicates that despite the desired mechanical properties similar to bone tissue or biocompatibility, PEEK is also characterized by biological inertness, making obtaining a proper connection between the bone and the PEEK implant impossible [16].

Advanced work on improving the properties of PEEK resulted in the creation of a glass-fiber reinforced composite. PEEK GRF is potentially another material which will be used in medical applications in future, which creates a necessity of carrying out appropriate research to evaluate its influence on the tissues after its implantation [9]. The material reinforced with 30% glass fiber (PEEK GRF30) is of particular interest. A large part of the studies are evaluations of bioactivity of pure PEEK or its carbon-reinforced (PEEK CRF) modifications [17,18,19,20]. However, there is no comprehensive research on PEEK GRF. This indicates the need to carry out analyses confirming or negating the functionality of this composite in terms of its biomedical applications.

The above information prompted the authors to conduct comprehensive PEEK GRF30 and Ti6Al4V SLM bioactivity studies in a controlled environment using SBF and HBSS as well as Ti6Al4V samples as a reference material. These studies, with the use of two different simulation fluids and materials not yet tested for bioactivity, are the basis for further, more detailed research.

## 2. Materials and Methods

In order to conduct the research, 10 cylindrical samples of PEEK GRF30, Ti6Al4V SLM, and wrought Ti6Al4V with a height of 3 mm and diameter of 10 mm were prepared. PEEK GRF30 (MCP Engineering Plastics Limited, Essex, UK) samples were cut from a rod made by injection molding (melt temperature = 370 °C, mold temperature = 170 °C, injection pressure = 14 MPa, secondary holding pressure = 10 MPa, and screw speed = 50 rpm [18]), reinforced with randomly distributed fibers with an approximate diameter of 10 µm. Chemical composition of fibers is as follows (wt.%): SiO_2_ = 60.0%, Al_2_O_3_ = 24.4%, CaO = 9.0%, MgO = 6.0%, Na_2_O = 0.5%, and K_2_O = 0.1% [19].

Ti6Al4V SLM samples were melted with appropriate laser beam parameters (diameter = 0.10 mm, power = 190 W, speed = 500 mm/s, energy density = 127 J/mm^3^, and porosity < 0.5%). The layer thickness was 0.03 mm. At the end of the melting process, the samples were heat treated (850 °C for two hours; EOSINT M280, (EOS company, Krailling, Germany).

The rod made of biomedical forged Ti6Al4V alloy (American Elements, Los Angeles, CA, USA) was cut with a blade made of sintered carbides (H.B. Carbide, Lewiston, MI, USA).

**Figure 1:**
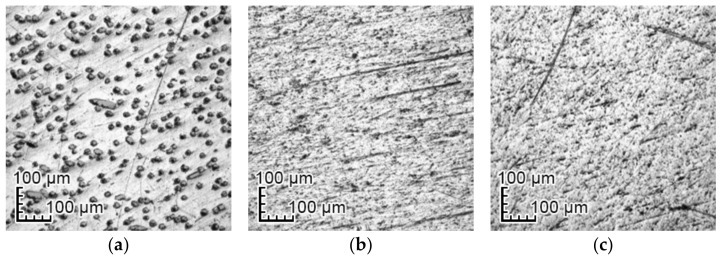
Examples of surface condition after polishing: (**a**) PEEK GRF30 (reinforced with 30% glass fiber), (**b**) Ti6Al4V SLM (selective laser melting), and (**c**) Ti6Al4V.

The surfaces of all samples were polished using a polishing cloth and gamma-type alumina polishing powder with a grain size of 0.05 μm. Average surfaces roughness, calculated on the basis of 10 measurements equally distributed profile lines, were as follows: PEEK GRF30: Ra = 0.207 ± 0.046 µm, Ti6Al4V SLM: Ra = 0.192 ± 0.024 µm, and Ti6Al4V: Ra = 0.155 ± 0.014 µm. The microscopic photos showing the surface of the samples are shown in Figure 1.

The samples were purified in 70% ethanol using an ultrasonic washer, rinsed with deionized water, and then, the samples were sterilized. The risk of microbial contamination was reduced by briefly immersing the samples in 70% isopropanol and rinsing with deionized water, followed by steam sterilization at 121 °C for 20 min (Systec VX-VE, Systec GmbH Labor-Systemtechnik, Linden, Germany).

The study was conducted in accordance with the relevant ISO 23317:2014 standard [21]. In order to carry out this work, two different simulation fluids were used—HBSS (Hank’s Balanced Salt Solution) and SBF (Simulated Body Fluid). To create the simulation fluids, reagents of high quality and purity were used (Macco Organiques, Zahradní, Czech Republic). The chemical composition of the proposed solutions (presented in Table 1) was selected so that the concentration of ions was comparable to the concentration of biological fluids, and the pH value (verified with the use of pH-meter FEP30 FiveEasy Plus™, Mettler Toledo, Columbus, OH, USA with an electrode Clarytrode 120, Hamilton, Giarmata, Romania) was as close as possible to the pH of human blood.

After preparation, the solutions were placed in glass flasks in a laboratory refrigerator maintaining a temperature of 7 °C for 24 h. After this time, the solution of a volume of 100 mL was poured into sterile containers. Individual samples were placed in each of these containers. The containers were then placed in an incubator at 37 °C and removed individually after 2, 7, 14, 21, and 28 days. The samples were dried in open air and gently wiped with sheets of paper towel. The surface topography of the samples was assessed using a HITACHI S-3000N scanning electron microscope (Hitachi High-Tech Science America, Inc, Chatsworth, CA, USA). Ti6Al4V SLM and Ti6Al4V samples were tested in high vacuum using a secondary electrons (SE) detector, while PEEK GRF30 in a low vacuum and with a backscattered electrons (BSE) detector.

The use of high vacuum and SE detectors allowed one to obtain higher resolutions, however, they can only be used effectively for conductive materials. For this reason, it was necessary to use a low vacuum and BSE detectors to test non-conductive PEEK GRF30 [22]. The chemical composition of the layer formed on the surface of the samples was evaluated using the Energy Dispersive Spectroscopy (EDS) analyzer (Hitachi High-Tech Science America, Inc, Chatsworth, CA, USA).

## 3. Results

### 3.1. Optical Changes in the Morphology of Layers Formed on the Surface

The first stage of the research was the optical analysis of changes in the morphology of the layers formed on the surface of the tested samples. The adopted test methodology included testing a new sample each time, because the one that was taken out of the simulation fluid was simultaneously exposed to air and then to a vacuum.

**Figure 2:**
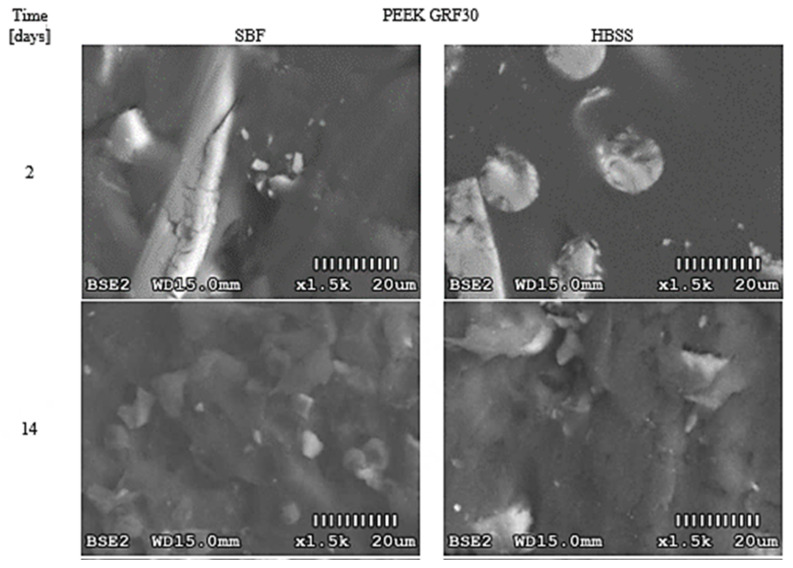

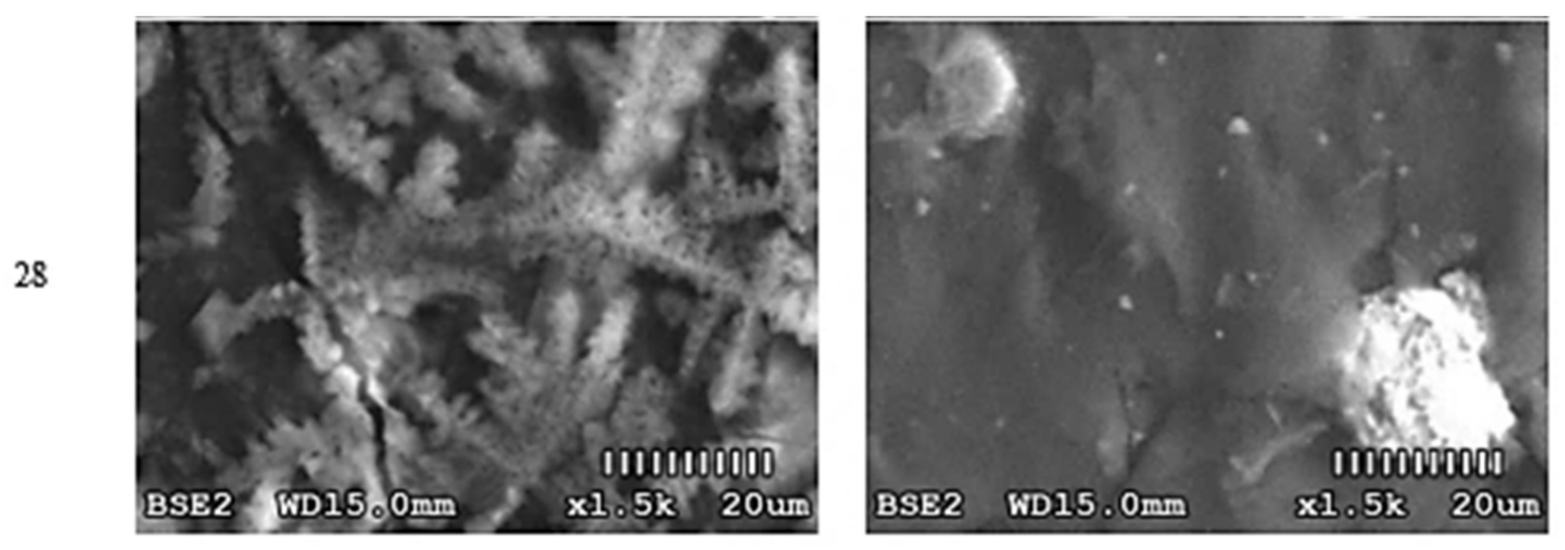
Morphological changes on the surface of PEEK GRF30 immersed in Simulated Body Fluid (SBF) and Hank’s Balanced Salt Solution (HBSS) after selected time periods.

**Figure 3:**
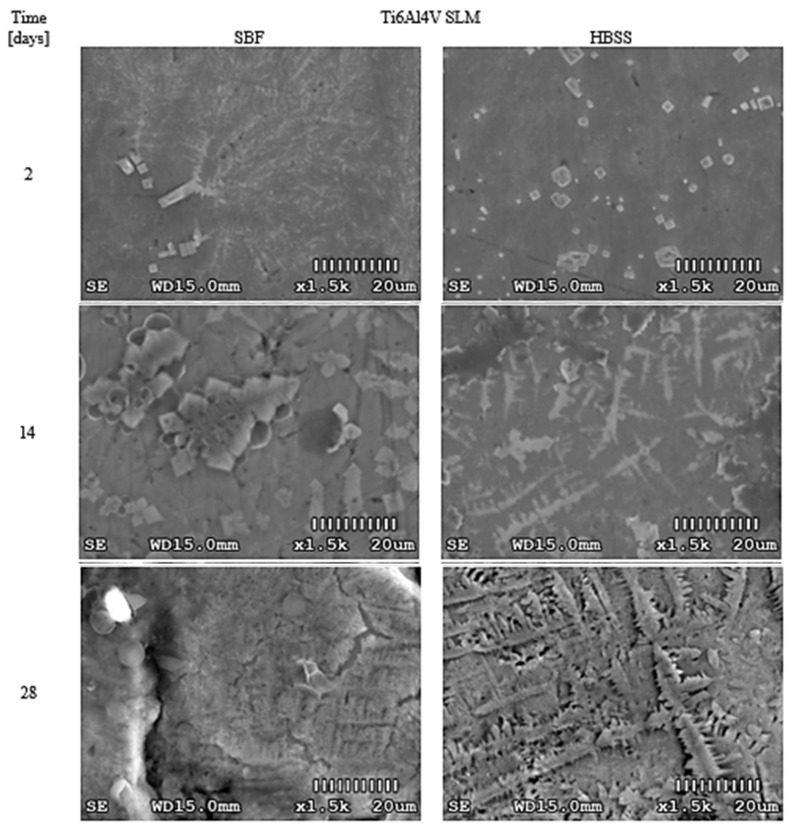
Morphological changes on the surface of Ti6Al4V SLM immersed in SBF and HBSS after selected time periods.

**Figure 4:**
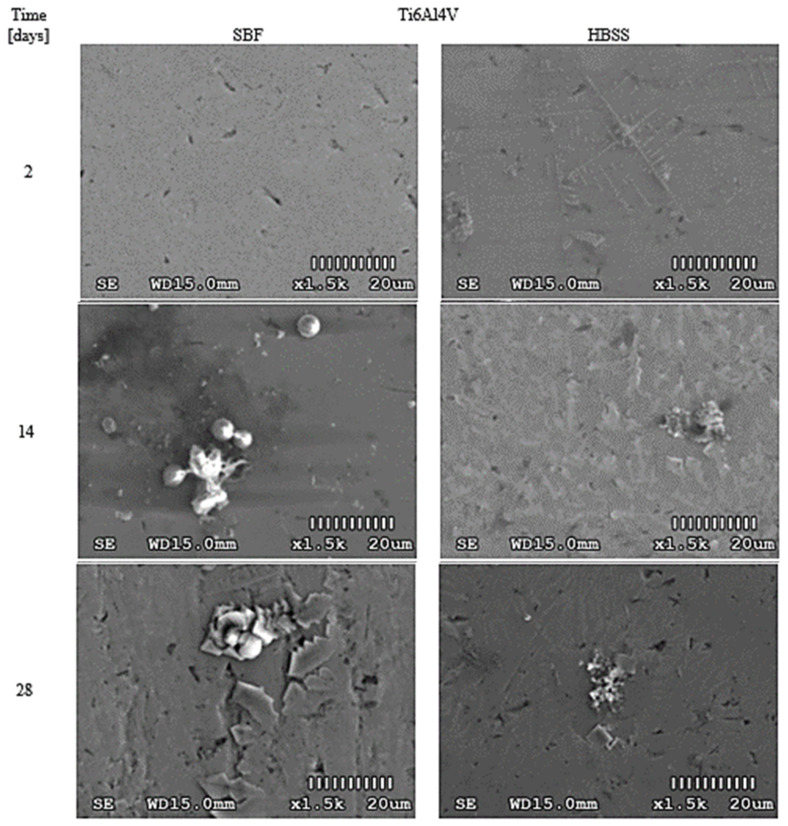
Morphological changes on the surface of Ti6Al4V immersed in SBF and HBSS after selected time periods.

To increase the objectivity of assessments, surface photos were taken at the same magnification (1500×). The obtained results for PEEK GRF30, Ti6Al4V SLM, and Ti6Al4V are presented in Figure 2, Figure 3 and Figure 4, respectively. The influence of the applied solutions on the morphology of the created layers can be noticed. In all samples, the use of SBF resulted in cross-partitions which formed a continuous layer after 14–21 days. This regularity was observed for all analyzed samples. However, in some cases, randomly separated nodular formations were noted. In contrast to SBF, the use of HBSS resulted in leaf-like partitions with visible sharp edges. Over time, these separations also formed a continuous layer, however, with noticeable irregularities.

### 3.2. Changes in the Chemical Composition of the Surface

Due to the large number of results, the authors decided to limit the presented data to the chemical composition of the surface layers of the samples before the test and after 28 days of immersion in SBF and HBSS solutions.

**Figure 5:**
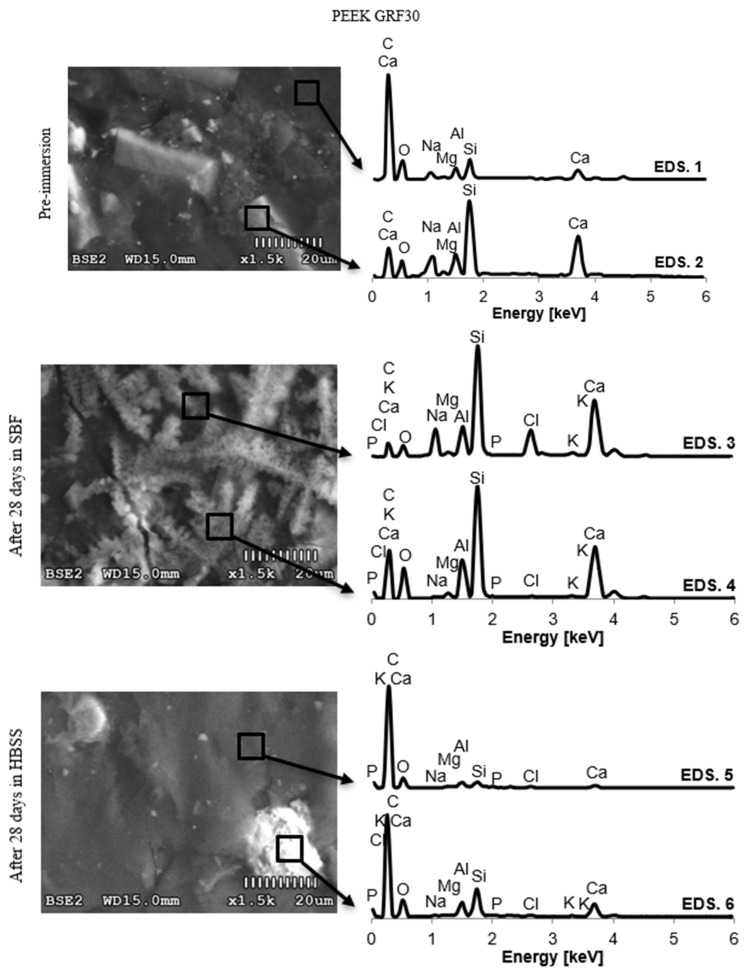
Spectrum of Energy Dispersive Spectroscopy (EDS) of PEEK GRF30 before and after 28 days of material samples immersion in SBF and HBSS.

**Figure 6:**
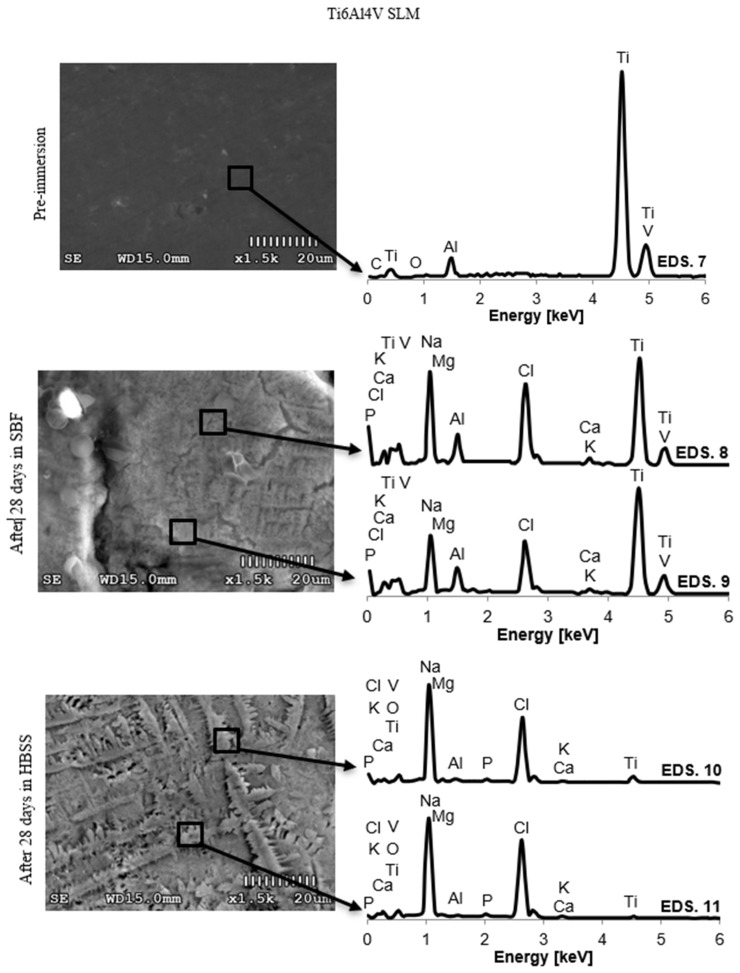
Spectrum EDS of Ti6Al4V SLM before and after 28 days of material samples immersion in SBF and HBSS.

**Figure 7:**
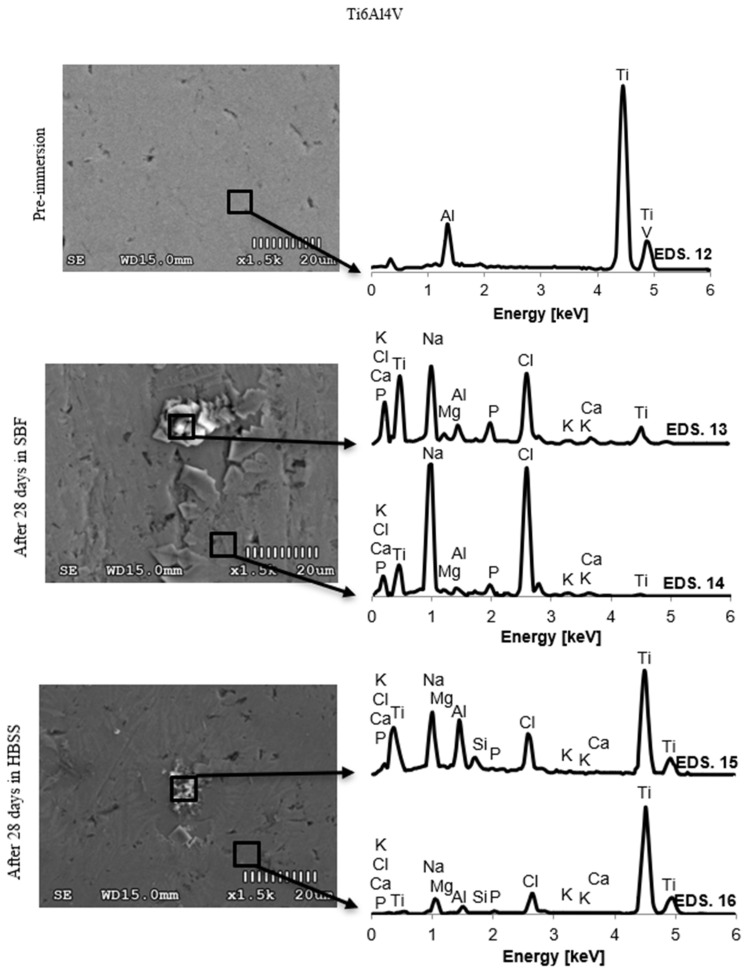
Spectrum EDS of Ti6Al4V before and after 28 days of material samples immersion in SBF and HBSS.

The obtained results describing the changes in the EDS spectrum of PEEK GRF30, Ti6Al4V SLM, and Ti6Al4V samples are shown in Figure 5, Figure 6 and Figure 7, respectively. The chemical composition of the formed crystals and precipitates is presented in Table 2. Immersion in SBF and HBSS solutions adsorbed phosphate ions on the sample surface, which then absorbed calcium ions to form calcium phosphate. Over time, the Ca/P ratio increased, which was confirmed in further studies.

## 4. Discussion

The assessment of the osteoinduction of medical material is the basis for further evaluation of its long-term functionality [4,6,8]. For this reason, the in vitro study of the possibility of hydroxyapatite formation on PEEK GRF30 and Ti6Al4V SLM also allows one to evaluate the functionality of appropriate orthopedic implants, spine implants, craniofacial implants, or implants for direct skeletal attachment of a limb prosthesis.

### 4.1. Optical Analysis of Changes on the Surface

**Figure A1:**
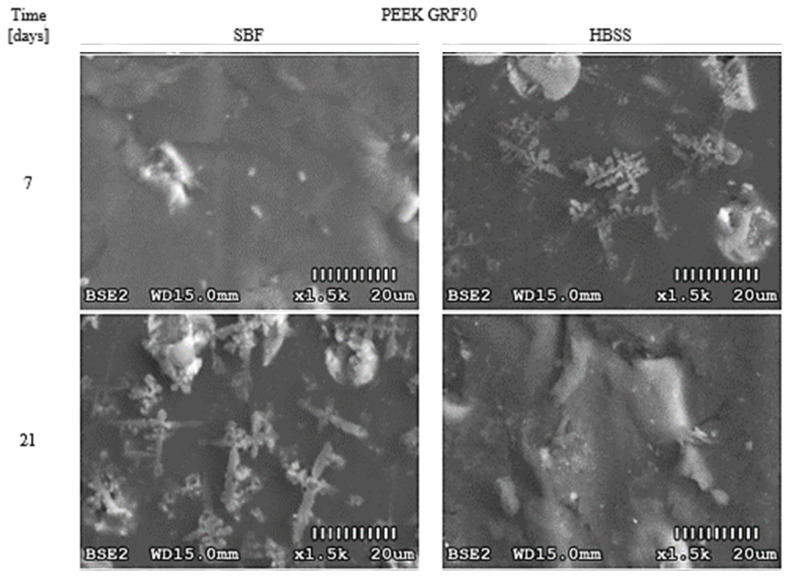
Morphological changes on the surface of PEEK GRF30 immersed in SBF and HBSS after selected time periods.

In carrying out image analysis, the different morphology of the layers formed on the test materials can be seen. In addition, the type of solution used also influenced the morphology of the resulting formations. In the case of PEEK GRF30 immersed in HBSS solution, cross-precipitation was observed 7 days after immersion, which, however, appeared only on a small area of the sample surface (Appendix A, Figure A1).

At the same time, no changes were observed on the sample immersed in the SBF solution. In the HBSS solution, after 14 days, a slow formation of fine crystals is observed, while playing in SBF, these crystals are already much larger. Differences in the morphology of the outer layers formed in the given solutions are also visible after 21 days. The crystals formed in HBSS are characterized by a structure similar to that observed two weeks earlier on samples immersed in SBF. On the other hand, samples immersed in SBF after 21 days show a dense coating on the surface. In another test, after 28 days, spherical precipitates appeared on the surface, and the coating is visually more extensive compared to the layer formed in HBSS. Here, in turn, cross-separation was observed, which, however, did not cover the entire surface of the sample, but only selected areas. The obtained layers in both cases differed from those obtained by other researchers, including Peng et al. [22]. However, in their research they focused on the evaluation of the bioactivity of PEEK surface modifications and therefore cannot be a direct reference to the results obtained by the authors.

**Figure A2:**
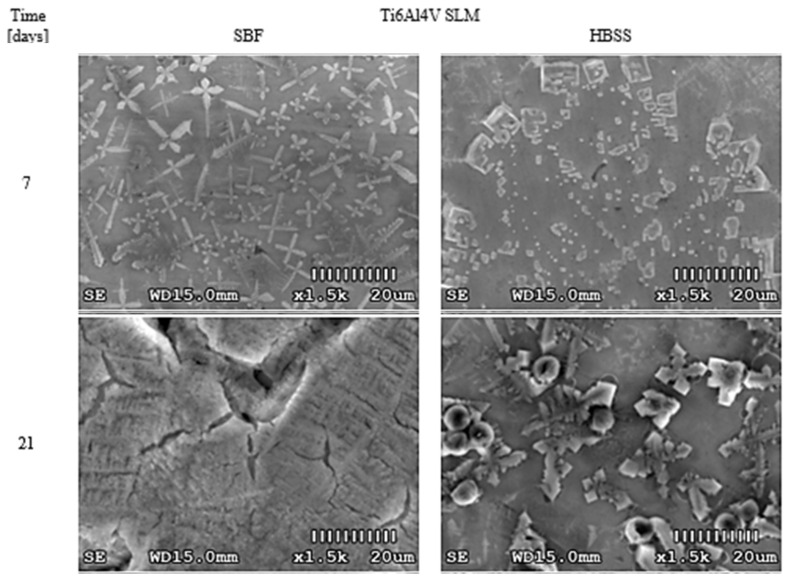
Morphological changes on the surface of Ti6Al4V SLM immersed in SBF and HBSS after selected time periods.

Nucleation of calcium phosphate crystals, both on the surfaces of Ti6Al4V SLM samples treated with SBF and HBSS solution (Figure 3, Appendix A, Figure A2), was observed after two days of immersion, which proves the high bioactivity of the tested surfaces. The morphology of the 20–30 nm crystals on both samples was similar, although a greater number of precipitates was observed on the sample immersed in the HBSS solution. The crystals isolated on the surface of this sample were dense, lumpy, and densely placed, while the crystals isolated from SBF were angular, did not form large clusters as in the case of HBSS, but very fine, regular, lamellar particles formed around them.

Subsequent optical surface studies, carried out after 7 and 14 days, revealed growth of crystals and change in their morphology. In the samples stored in the SBF solution, the crystals formed in the characteristic, not described before in the literature, cross-like shapes that formed large and numerous clusters. After 7 days on the surface of the samples immersed in the HBSS solution, further growth of the crystals was observed, the shape of which changed from spherical to more compact and denser. There were also lamellar secretions similar to those found on SBF samples after 2 days. After 14 days, the spheroidal precipitates were also observed on the HBSS samples, the number of which multiplied in the following days. After 21 days, the subsequent immersion of the sample placed in the SBF solution caused the surface to be covered with a cracked, heterogeneous, and rough layer, which in the next days increased, forming a uniform shell with spherical sections disseminated on it. After 28 days, on the surface of the sample immersed in HBSS solution, hydroxyapatite formed a compact coating with a strongly developed texture.

**Figure A3:**
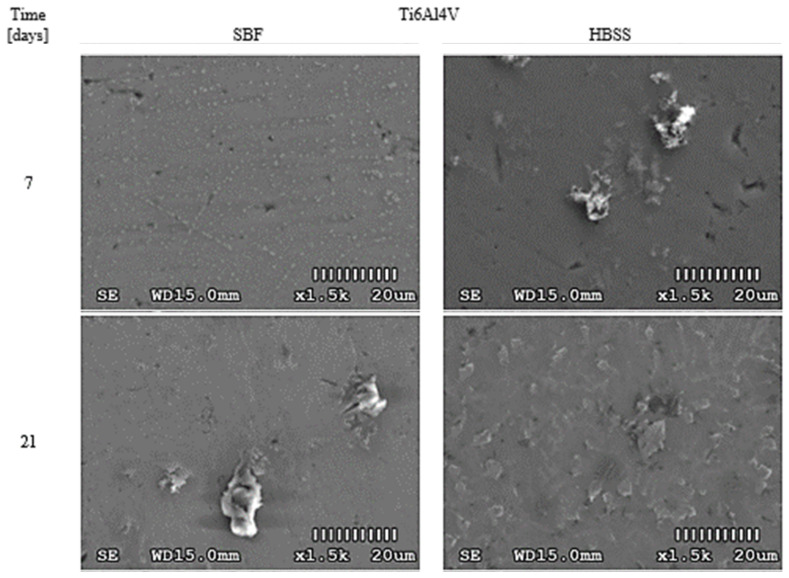
Morphological changes on the surface of Ti6Al4V immersed in SBF and HBSS after selected time periods.

In the case of optical analyses of Ti6Al4V samples (Figure 4, Appendix A, Figure A3), it can be seen that after 7 days, relatively small and spheroidal structures formed on the surface of the samples immersed in SBF, the size of which increased significantly after 14 days. Then, after 28 days, further intensification of the crystal growth process was observed. The structure of the obtained crystals is similar to the formations obtained by Yoshida and Hayakawa, among others, who also tested Ti6Al4V immersed in SBF solution [12]. In the case of the Ti6Al4V samples in HBSS, after 2 days, small amounts of crystals in the form of elongated aggregates were observed. A change in this structure was observed in the following days. In this case, the obtained layers also resembled those presented in the literature by Lu et al. [13], among others.

Immersion in SBF and HBSS solutions in a short time resulted in adsorption to the surface of the samples of phosphate ions, which then absorbed calcium ions, forming amorphous calcium phosphate. The increase in the concentration of Ca^+2^ ions was due to the electrostatic attraction of negatively charged TiOH- groups located on the implant surface. The increase in the concentration of calcium ions resulted in exceeding the solubility product for calcium phosphate and an increase in pH above 8 (in the HBSS solution on the second day and in the SBF solution on the third day), which favored the formation of hydroxyapatite [23]. As a result, the Ca/P ratio increased over time, which was confirmed in further studies. Amorphous calcium phosphate, due to the addition of calcium and phosphorus ions from the SBF solution, formed on the samples immersed in the HBSS solution, formed a compact, although not uniform in terms of morphology coating, and the pH stabilized after twenty-one days from the start of the study. In turn, the hydroxyapatite layer turned into a dense, uniform apatite after about twenty days, which was confirmed by microscopic examinations and stabilized pH of the solution.

The bioactivity of the analyzed metallic materials, i.e., Ti6Al4V SLM and Ti6Al4V, is most likely the result of the ability of the titanium alloy to spontaneously form an oxide layer when exposed to air. This layer, apart from increasing corrosion resistance, also positively influences the formation of apatite on the surface of the material [24,25]. It should be emphasized, however, that in the case of Ti6Al4V, unlike the Ti6Al4VSLM samples, there was no continuous, unbroken layer of hydroxyapatite, but only local precipitation that did not cover the entire surface of the tested samples. In the case of PEEK GRF30, despite reports of bioinertia of its pure form, pores or unevenness resulting from the reinforcement may increase the contact surface with the deposited layer of hydroxyapatite [26,27]. The same factor may also promote the formation of apatite layers in the case of Ti6Al4V SLM, where the pores formed during the powder melting process also increase the total surface area of the sample.

### 4.2. Changes in the Chemical Composition of the Surface

Scanning electron microscopy with the EDS (Energy Dispersive Spectroscopy) system is a research method used in material research to analyze (morphology and elemental composition) of the surface layer of the tested materials. A specific area of the surface of the test sample was subjected to the action of a concentrated electron beam, which penetrated the surface layer of the material. The secondary electron signal and the radiation energy value were collected by the EDS detector, then processed and displayed in the form of a spectrum. The value of the radiation energy is characteristic of various elements, and the height of the peaks indicates their intensity.

The morphological changes of the formed coatings were accompanied by changes in the chemical composition on the surfaces of the tested samples. The mass share of individual elements in the precipitates formed on the sample surfaces and the graphical representation of the concentration changes of selected chemical elements after twenty-eight days of immersion are presented in Table 2 and Figure 5, Figure 6 and Figure 7.

The results of changes in the content of the outer layer in the case of PEEK GRF30 are difficult to interpret due to the chemical composition of glass fibers, which include elements characteristic of hydroxyapatite (in this case Ca) and other inorganic compounds (including Mg), which accumulate on the surface of the sample during immersion in body fluids. The interaction of the electron beam on the surface gave different results each time, which clearly indicates that the chemical composition of the matrices differed significantly from the chemical composition of glass fibers (therefore, in Figure 5 of the samples before immersion, two spectra are presented). Nevertheless, after 28 days, there was a significant increase in the content of Ca and P and Na, Cl, and K in the PEEK GRF30 surface exposed to the SBF solution. At the same time, the decrease in the intensity of the carbon (C) peaks, which is the main component of the PEEK polymer, suggests that the apatite layer tightly covered the surface of the sample. In the case of analyses of changes in the chemical composition of PEEK GRF30 samples immersed in HBSS, a slight content of P, Cl, and K was noted, and after 28 days, no significant changes in the chemical composition were observed. The content of detected C and O is close to the initial value, which may suggest that the sample surface is penetrated by the scanning beam of the microscope without any obstacles. This, in turn, confirms the observations that PEEK was not covered with a continuous apatite layer.

Results obtained for Ti6Al4V SLM indicate that Cl, P, Cl, and K were deposited on the analyzed areas in a substantial amount, while Mg and Na was in slightly less amount. Higher levels of Ca and P were obtained on samples stored in SBF solution. The average value of the Ca/P ratio for samples stored in the HBSS solution was approx. 1.52, while for samples stored in the SBF solution 1.62. Such a high stoichiometric ratio of the precipitated samples formed on the surfaces indicates convergence with calcium phosphate Ca_3_(PO_4_)_2_. (Ca/P ratio = 1.5) and hydroxyapatite (Ca/P ratio = 1.67). The layer growing on the surface of the samples influenced the change in the transmission of the scanning beam, whereby the detected concentration of the basic elements of the substrate (Ti, Al, V) was lower with each subsequent test.

When analyzing the EDS results obtained from scanning the surface of samples made of Ti6Al4V and immersed in SBF, it can be concluded that the visible precipitates consisted of Ca and P. The concentration of these elements in the precipitates was greater than around them. At the same time, it was observed that the concentration of the mass fraction of the main elements, such as Ti, Al and V in each subsequent test was lower, which proves that the surface of the sample was covered with a thin apatite layer. In the case of samples placed in HBSS, the concentration of elements forming hydroxyapatite—Ca, P, Na, and K—was much lower. The regularity observed in the case of samples immersed in SBF was again observed—the precipitates visible in the photos were clearly richer in these elements. The high content of Ti, Al, and V indicates that the outer layer has been almost completely penetrated by the scanning beam, suggesting that the formed layer is relatively thin. The ratio of Ca and P, in this case, is lower than 1.67—the average result is 1.52, which indicates the formation of calcium orthoposphate (V) compounds.

Although, the solutions had similar compositions, the concentrations of ions were different, which could affect the mechanisms of a hydroxyapatite deposition. In the case of HBSS, there was a higher concentration of SO_4_^2−^ ions, while in SBF the tris buffer was used along with slightly higher concentration of Ca^2+^ ions and high capacity of HCl. According to the data of the reference, the tris buffer used with HCl affect final composition of created spherical-like precipitates, which were also obtained, i.a. on presented PEEK GRF30 and Ti6Al4V SLM, immersed in SBF solution. Furthermore, higher concentration Ca^2+^ ions could provide higher possibility of their combination with PO_4_^3−^ ions, present in the solution. Higher concentration of SO_4_^2−^ with deficit of Ca^2+^ ions can result in forming sulfate-ion-substituted hydroxyapatite however, this phenomenon was not a matter of presented research.

Analyzing the research published so far, it can be noted that the most intensive research is conducted to verify bioactivity of titanium alloys. Although the methodology used in the research was often convergent, the obtained results were extremely different. For example, [28] obtained a thin, porous apatite film after just 30 min of interaction with SBF, while [29,30] observed the formation of such a layer only after 28 days. In none of the above articles, the structure of the precipitates resembled the apatite that appeared on the samples studied by the authors of this article.

Among the many articles describing the research using Ti6Al4V SLM, only a few dealt with the topic of osseointegration—most often the authors consider that the bioactivity of these alloys is the same as that of forged alloys. Few researchers conducted an attempt to verify this phenomenon and i.a. [31] obtained apatite crystals on anodized Ti6Al4V SLM samples after about two weeks, but these precipitations resembled the structure of crystals obtained on the Ti6Al4V surface after 2 days of immersion in HBSS. Also, few articles have been published on PEEK bioactivity. Interesting observations were shared by [32], who indicated that no single apatite crystals were formed on the surface of PEEK samples for 28 days. At the same time, the EDS analysis showed a decreasing trend for the content of Ca and P, which was also shown in the studies presented in this article. No article on PEEK GRF30 bioactivity has been published so far, which for now, makes it impossible to compare any results obtained.

## 5. Conclusions

Osteoinduction is the process by which stem cells differentiate and induce them to transform into osteoblasts, which leads to the rebuilding of bone tissue. The osteoinductive properties of the biomaterial are the result of its bioactivity—one of the key properties of the biomaterial, which in contact with blood plasma manifests itself through the ability to biomineralize the hydroxyapatite layer on the surface of the biomaterial, which is a scaffold for the reconstruction of bone tissue.

The formation of the apatite layer is important due to the improvement of the abrasion resistance of the implant material and the limitation of the penetration of undesirable compounds into the biological environment. The presence of a bioactive apatite coating ensures the fixation of the implant in the bone without the need for bone cement. The possibility of creating a permanent connection at the bone-implant interface was verified on the basis of in vitro tests using fluids simulating the physiological environment. The evaluation of the osteoinduction of Ti6Al4V, Ti6Al4V SLM, and PEEK GRF30 in two simulation solutions—SBF and HBSS revealed that apatite crystals appeared on the surface of the samples after two days as a result of phosphorus and calcium penetration. The positively charged calcium ions were incorporated into the hydrated TiOH^−^ layer, and then the negatively charged (PO_4_)^3−^ and (CO_3_)^2−^ were attached to them, creating the Ca/P surface layer, which, as a result of further ion adsorption, crystallized into bone-like apatite. The rate of crystallization was the result of high surface energy as well as microporosity and surface irregularities, which are an important factor in the nucleation of calcium and phosphorus ions. In addition, it has been reported that the kinetics of hydroxyapatite precipitation is faster when it is due to ion exchange between the surface and SBF.

The research proved that each of the tested materials has the potential to form apatite on the surface, which, however, only in the case of Ti6Al4V SLM formed a compact layer with the correct Ca/P ratio. The morphology of the new layer and EDS results showed that the layer correspond to apatite. The obtained results also indicate that there is a possibility of deposition of apatite on the surface of PEEK GRF30. This suggests that osseointegration can be achieved between the bone and the PEEK composite implant without the use of an additional porous layer on the implants, but the apatite layer formation time is much longer than in the case of a titanium alloy. It can therefore be concluded that PEEK GRF30 and Ti6Al4V SLM are characterized by bioactivity, providing implants with appropriate biofunctionality.

## Figures and Tables

**Table 1:** Chemical composition of solutions prepared for bioactivity tests.

HBSS pH = 7.3	Conc. *	SBF pH = 7.3	Conc. *
NaCl	0.140 M	NaCl	0.137 M
CaCl_2_	0.002 M	CaCl_2_	0.003 M
KCl	0.005 M	KCl	0.003 M
NaHCO_3_	0.004 M	NaHCO_3_	0.004 M
MgCl_2_∙6H_2_O	0.002 M	MgCl_2_∙6H_2_O	0.002 M
NaH_2_PO_4_	0.002 M	Na_2_SO_4_	0.001 M
MgSO_4_	0.002 M	K_2_HPO_4_ 3H_2_O	0.001 M
D-glucose	0.006 M	Tris	0.051 M
		1.0 M HCl	39.000 mL
		1.0 M HCl (added until pH = 7.3)	up to 5 mL

* Conc.—concentration.

**Table 2:** Atomic percentage of individual elements before and after 28 days of material immersion in SBF and HBSS.

		EDS	C	O	Na	Mg	Al	Si	P	Cl	K	Ca	Ti	V
PEEK GR30	Pre-immersion	1	68.63	26.21	1.32	0.22	0.96	1.86	0	0	0	0.79	0	0
			± 0.62	± 0.51	± 0.04	± 0.02	± 0.02	± 0.02				± 0.01		
		2	53.85	23.59	0.85	0.33	1.70	16.38	0	0	0	3.30	0	0
			± 0.70	± 0.36	± 0.09	± 0.05	± 0.04	± 0.05				± 0.04		
	SBF (28 days)	3	33.88	25.05	7.22	0.82	4.37	15.88	1.42	3.42	0.36	7.57	0	0
			± 0.46	± 0.56	± 0.11	± 0.05	± 0.08	± 0.11	± 0.03	± 0.03	± 0.02	± 0.08		
		4	46.97	34.59	0.31	0.46	3.09	9.24	1.14	0.10	0.14	3.95	0	0
			± 0.34	± 0.35	± 0.03	± 0.01	± 0.03	± 0.05	± 0.01	± 0.01	± 0.01	± 0.03		
	HBSS (28 days)	5	74.96	22.67	0.07	0.04	0.80	0.75	0.28	0.04	0.01	0.38	0	0
			± 0.48	± 0.41	± 0.02	± 0.02	± 0.02	± 0.01	± 0.01	± 0.01	± 0.01	± 0.02		
		6	68.02	25.69	0.14	0.15	1.34	2.75	0.50	0.09	0.04	1.27	0	0
			± 0.45	± 0.34	± 0.02	± 0.01	± 0.02	± 0.03	± 0.01	± 0.01	± 0.02	± 0.02		
Ti6Al4V SLM	Pre-immersion	7	0.23	0.54	0	0	10.09	0	0	0	0	0	85.52	3.62
			± 0.08	± 0.09			± 0.08						± 0.24	± 0.07
	SBF (28 days)	8	0	0.49	0.38	0.15	9.00	0	6.04	0.68	0.48	10.28	69.2	3.29
				± 0.05	± 0.14	± 0.13	± 0.10		± 0.06	± 0.02	± 0.06	± 0.14	± 0.44	± 0.16
		9	0	0.51	0.46	0.17	8.79	0.18	7.60	0.87	0.45	12.7	64.95	3.32
				± 0.07	± 0.14	± 0.11	± 0.13	± 0.05	± 0.04	± 0.19	± 0.03	± 0.09	± 0.52	± 0.12
	HBSS (28 days)	10	0	0.16	1.34	0.11	5.13	0	6.72	0.96	0.17	10.8	72.27	2.34
				± 0.11	± 0.02	± 0.04	± 0.09		± 0.14	± 0.07	± 0.05	± 0.11	± 0.32	± 0.07
		11	0	0.13	1.09	0.14	4.80	0	7.05	0.1	0.19	11.8	72.02	1.90
				± 0.05	± 0.13	± 0.03	± 0.16		± 0.05	± 0.12	± 0.06	± 0.08	± 0.27	± 0.09
Ti6Al4V	Pre-immersion	12	0	0	0	0	9.02	0	0	0	0	0	88.04	2.94
							± 0.10						± 0.46	± 0.15
	SBF (28 days)	13	0	0	4.12	0.50	6.4	0	5.81	3.2	1.58	8.42	67.52	2.45
					± 0.41	± 0.14	± 0.09		± 0.11	± 0.55	± 0.14	± 0.31	± 0.22	± 0.03
		14	0	0	2.15	1.52	6.80	0	5.67	2.73	1.64	8.07	68.29	3.13
					± 0.37	± 0.18	± 0.14		± 0.16	± 0.50	± 0.14	± 0.20	± 0.66	± 0.09
	HBSS (28 days)	15	0	0	1.2	0.24	8.11	0.91	4.72	0.70	0.76	7.19	73.22	2.95
					± 0.08	± 0.02	± 0.04	± 0.10	± 0.16	± 0.13	± 0.07	± 0.06	± 0.36	± 0.09
		16	0	0	1.0	0.76	8.14	0.62	4.57	0.74	1.24	7.21	72.57	3.05
					± 0.04	± 0.14	± 0.04	± 0.17	± 0.11	± 0.05	± 0.12	± 0.07	± 0.31	± 0.07

## Data Availability

The data presented in this study are available on request from the corresponding author.
